# Enhancing Clinician Trust in AI Diagnostics: A Dynamic Framework for Confidence Calibration and Transparency

**DOI:** 10.3390/diagnostics15172204

**Published:** 2025-08-30

**Authors:** Yunguo Yu, Cesar A. Gomez-Cabello, Syed Ali Haider, Ariana Genovese, Srinivasagam Prabha, Maissa Trabilsy, Bernardo G. Collaco, Nadia G. Wood, Sanjay Bagaria, Cui Tao, Antonio J. Forte

**Affiliations:** 1 Zyter|TruCare, Rockville, MD 20852, USA; 2 Division of Plastic Surgery, Mayo Clinic, Jacksonville, FL 32224, USA; 3 Department of Radiology AI IT, Mayo Clinic, Rochester, MN 55905, USA; 4 Division of Surgical Oncology, Mayo Clinic, Jacksonville, FL 32224, USA; 5 Department of Artificial Intelligence and Informatics, Mayo Clinic, Jacksonville, FL 32224, USA; 6 Center for Digital Health, Mayo Clinic, Rochester, MN 55905, USA

**Keywords:** AI decision support systems, clinician trust, confidence calibration, AI transparency

## Abstract

**Background:** Artificial Intelligence (AI)-driven Decision Support Systems (DSSs) promise improvements in diagnostic accuracy and clinical workflow efficiency, but their adoption is hindered by inadequate confidence calibration, limited transparency, and poor alignment with real-world decision processes, which limit clinician trust and lead to high override rates. **Methods:** We developed and validated a dynamic scoring framework to enhance trust in AI-generated diagnoses by integrating AI confidence scores, semantic similarity measures, and transparency weighting into the override decision process using 6689 cardiovascular cases from the MIMIC-III dataset. Override thresholds were calibrated and validated across varying transparency and confidence levels, with override rate as the primary acceptance measure. **Results:** The implementation of this framework reduced the override rate to 33.29%, with high-confidence predictions (90–99%) overridden at a rate of only 1.7%, and low-confidence predictions (70–79%) at a rate of 99.3%. Minimal transparency diagnoses had a 73.9% override rate compared to 49.3% for moderate transparency. Statistical analyses confirmed significant associations between confidence, transparency, and override rates (*p* < 0.001). **Conclusions:** These findings suggest that enhanced transparency and confidence calibration can substantially reduce override rates and promote clinician acceptance of AI diagnostics. Future work should focus on clinical validation to optimize patient safety, diagnostic accuracy, and efficiency.

## 1. Introduction

Due to its constant and ever-evolving nature, Artificial Intelligence (AI) is now recognized for its potential to enhance healthcare, particularly through Decision Support Systems (DSSs). These systems leverage extensive data and sophisticated algorithms to support clinicians by improving diagnostic accuracy, personalizing treatment decisions, and ultimately enhancing patient outcomes [1,2]. However, despite this promise, AI integration into routine clinical practice remains limited. Key barriers to its adoption include insufficient transparency, inconsistent confidence calibration, and challenges in defining clear override mechanisms that effectively balance AI suggestions with clinician expertise [1,3].

Transparency in AI models is essential for gaining clinician trust and enabling effective collaboration. Explainable AI (XAI) aims to clarify how AI systems arrive at decisions, thus helping clinicians feel more confident in AI-generated recommendations [4]. Nevertheless, many current AI models remain opaque, fostering skepticism and hesitation among healthcare providers. These findings highlight the complexity of ensuring that AI transparency aligns effectively with clinical needs.

Confidence calibration is equally vital for ensuring AI reliability in clinical settings. AI systems that are poorly calibrated may either overestimate or underestimate their diagnostic accuracy, resulting in misdiagnoses, unnecessary medical interventions, or overlooked critical conditions. A recent study clearly demonstrated this issue, revealing that AI models missed approximately 66% of life-threatening injuries in hospitalized patients, underscoring the urgent need for robust calibration procedures [5]. Additionally, stochastic neural networks have been found to inadequately represent epistemic uncertainty, creating a false impression of confidence and limiting their reliability for clinical decision support [6,7].

Determining appropriate override thresholds—when clinicians should disregard or adjust AI recommendations—is also essential to patient safety and clinical effectiveness. Poorly defined override strategies risk either excessive reliance on AI, potentially neglecting nuanced clinical insights, or frequent overrides, diminishing the system’s practical utility. For example, a recent study on AI-supported breast cancer screening indicated that although high confidence scores from AI systems boosted clinician trust, they also led to over-reliance, negatively impacting diagnostic accuracy. Conversely, low AI confidence reduced trust, leading to longer decision-making times and cautious clinical behavior [8]. AI systems initially intended to lower healthcare costs often end up requiring substantial human oversight, illustrating the complexities involved in integrating AI effectively into clinical workflows without sacrificing clinician autonomy or patient safety [9,10].

Despite these barriers, AI’s benefits in healthcare are increasingly evident. Studies have successfully employed AI to diagnose pneumonia and COVID-19 using lung ultrasound imagery [11,12], detect diabetic retinopathy from retinal fundus images [13], and correctly classify benign versus malignant skin lesions [14], highlighting significant potential in diagnostic applications. To fully realize these benefits, however, the challenges of transparency, confidence calibration, and override mechanisms must be systematically addressed.

This study presents a novel scoring framework designed to dynamically adjust override thresholds based on AI confidence levels, semantic diagnosis similarity, and transparency. By integrating these factors, our approach seeks to improve clinician trust, precision, and practical utility of AI-generated diagnostics. Unlike traditional models that rely solely on fixed confidence scores, our dynamic, multifactor framework aims to reduce unnecessary clinician overrides, thereby facilitating greater acceptance and safer clinical adoption of AI technologies.

Recent work using the MIMIC-III database has established strong baselines for ICU decision support while underscoring the need for operational criteria that govern when to accept or override AI outputs. In heart-failure cohorts, machine learning models—including XGBoost, LASSO, and ensembles—have shown good discrimination and calibration for in-hospital mortality and have been translated into nomogram-style tools for bedside use [15,16]. Related efforts have addressed ICU discharge prediction for cardiovascular patients, reporting high accuracy and highlighting features linked to readiness for transfer, though without specifying acceptance/override rules for deployment [17]. In sepsis, models trained on MIMIC-III generally outperform traditional severity scores (e.g., SAPS II) and show better calibration in clinically relevant ranges, reinforcing the value of data-driven prediction for high-risk decisions [18]. More recently, comparisons between domain-specific transformers and general-purpose large language models on early emergency-department notes reveal a precision–recall trade-off, with transformers often leading on F1 while LLMs show higher recall, illustrating why predictive accuracy alone is insufficient without explicit, auditable logic for clinician acceptance or override [19]. Together, these studies motivate pairing predictive performance with transparent, thresholded decision rules that align AI suggestions with clinical judgment.

This work adds significant contributions to the current available literature on AI transparency mechanisms, including a dynamic, multi-factor acceptance rule that integrates model-reported confidence, semantic similarity, and a length prior with transparency-conditioned thresholds that tighten when explanations are weaker—an explicit mechanism to calibrate clinician reliance on AI outputs; a formal scoring formulation that defines a composite trust score and a single-step acceptance rule, enabling transparent implementation and auditability; operational use of transparency as a gate modifier rather than a stand-alone predictor, so high self-ratings cannot compensate for low confidence or low similarity; a priori, transparency-specific parameters (weights and thresholds) fully specified for straightforward deployment and review; pragmatic text-quality constraints via a minimum similarity cut-point (0.10) and a length prior that gives full credit to 30–100-character diagnoses to discourage vague or list-like outputs; a large-cohort evaluation on 6689 MIMIC-III cardiovascular cases demonstrating a reduction in overrides from 87.64% to 33.29%, emphasizing workflow-relevant impact beyond discrimination metrics; stratified evidence that confidence is the dominant driver of acceptance while transparency and similarity chiefly shape borderline decisions; statistical robustness through 95% confidence intervals for overall and stratified override rates alongside association testing (χ^2^ with Cramér’s V); and a full interaction analysis across confidence-by-transparency strata that clarifies when transparency meaningfully shifts decisions versus when confidence predominates.

## 2. Methods

### 2.1. Data Source

A cohort consisting of patients diagnosed with cardiovascular diseases (CVD) was built by extracting their data from the Medical Information Mart for Intensive Care III database. MIMIC-III is a publicly available, de-identified dataset containing comprehensive clinical information of over 60,000 patients admitted to the Beth Israel Deaconess Medical Center in Boston, Massachusetts, between 2001 and 2012. It includes a wide range of patient data, including demographics, laboratory tests, imaging reports, clinical notes, discharge notes, and ICD-9 codes [20].

CVDs were identified using ICD-9 codes relevant to ischemic heart disease (IHD), heart failure (HF), arrhythmias, cardiomyopathies (CMP), valvular heart diseases (VHD), and hypertensive heart disease (HTA). Table 1 presents the list of conditions included in our dataset, along with their respective ICD-9 codes, and Supplementary Table S1 shows the complete list of diagnoses as used in diagnoses_icd. Patients with multiple CVD diagnoses within a single admission were treated as unique cases. The cohort was restricted to adult patients (≥18 years) with at least one documented discharge summary in the *noteevents* document to ensure the availability of clinical documentation. This resulted in a total of 6689 cases being included in our analysis.

### 2.2. Data Preprocessing

Clinical notes and discharge summaries underwent standard text preprocessing, normalization, and deidentification to remove patient-identifiable information and irrelevant content. This also included converting text to lowercase, eliminating punctuation and unnecessary symbols, breaking text into individual words, filtering out common but non-essential words, and standardizing terms to their base forms for consistency.

### 2.3. Computational Framework

**Figure 1:**
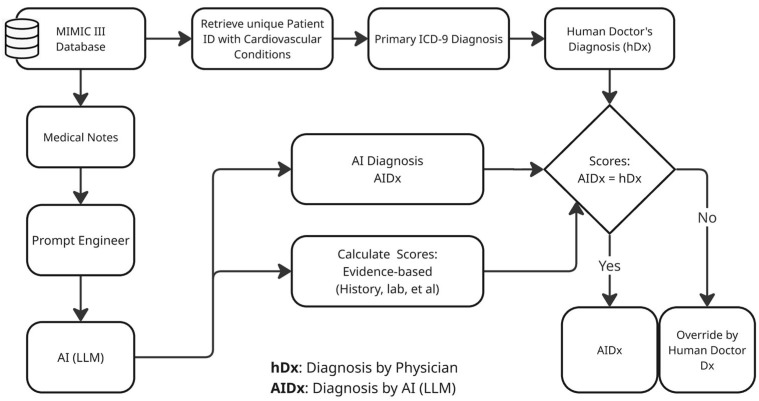
Schematic diagram illustrating the process of computing AI-generated diagnoses (AIDx) and comparing them with human physician diagnoses (hDx). The diagram depicts the end-to-end workflow, including data retrieval, filtering and preprocessing, AI diagnosis generation, and the evaluation of alignment with clinician-verified diagnoses (Dx). The evidence-based scoring system combines confidence ($w_{conf}$), similarity ($S_{sim}$), and length ($S_{len}$) to generate the final compound confidence scores to determine whether AIDx is trustworthy or not.

AI-generated diagnoses and true diagnoses were extracted from the *noteevents* and *diagnoses_icd* tables (provided upon request). Diagnosis similarity was computed using a combination of exact string matching and semantic similarity using the Universal Sentence Encoder (USE) model [21]. Figure 1 presents a schematic representation of our framework.

To generate the AI diagnoses (AIDx), the large language model (LLM) Qwen 2.5 was used off-the-shelf without additional fine-tuning. Inference settings were set as follows: temperature 0.1, nucleus sampling (top-p) 0.9, and a maximum of 256 generated tokens per diagnosis; stop sequences prevented run-on text. No gradient-based training was performed. The generated diagnoses were compared with the true diagnoses documented in the discharge summaries provided from the MIMIC-III dataset (hDx) (Figure 1). The decision to override AI-generated diagnoses was based on a scoring framework combining three key factors. First, *AI Confidence*, which was generated by the LLM in response to a structured prompt asking it to assess the strength of diagnostic evidence based on clinical cues such as vital signs, laboratory markers, organ dysfunction, temporal progression, comorbidities, and supporting documentation in the discharge summary. A confidence range of 70% to 99% was determined to automatically override very unreliable responses (<70%) while avoiding overconfident guessing. Therefore, this score reflects how certain the model is of its diagnosis, based on its internal interpretation of the clinical context and not on probabilistic outputs. The second factor considered was *Diagnosis Similarity*, determined by the semantic similarity score between the AIDx and the hDx and computed using cosine similarity of USE embeddings. Finally, the *Transparency Level* was obtained by instructing the model to rate the clarity and explainability of its diagnoses based on a three-point ordinal scale—low, moderate, and high—and served only to adjust the acceptance (override) threshold while never acting as an independent factor. These levels reflect how well the LLM could articulate its reasoning based on the clinical evidence provided. This structured self-assessment was embedded in the model’s output, allowing us to consistently code and analyze transparency as a categorical variable. While it is a self-rated metric by the LLM, it provides a standardized proxy for perceived explainability in the absence of a human rating for each prediction.

The scoring system was defined as Equation (1):

(1) $$S_{final}=w_{c}\times S_{conf}+w_{s}\times S_{sim}+w_{l}\times S_{len}$$

where

$S_{conf}$—AI Confidence score, normalized to a value between 0.5 and 1.0.

$S_{sim}$—Diagnosis Similarity score, with a minimum threshold of 0.1.

$S_{len}$—Length-based score, assigned as 1.0 for diagnoses between 30 and 100 characters, and 0.7 otherwise.

$w_{c},w_{s},w_{l}$—Weights assigned to confidence, similarity, and length based on the transparency level.

The scoring system includes response length—the diagnosis provided—as a proxy for information sufficiency and clarity, as in clinical contexts, extremely short AI-generated diagnoses may be too vague or non-actionable, while overly long or verbose ones may indicate uncertainty or overgeneration [6,8]. This parameter, therefore, allows for rewarding concise and specific diagnoses and penalizing outputs that are either too short to be useful or too verbose to be practical. Moreover, we have observed that well-formed diagnoses in our dataset often fall within the 30–100-character range. Nevertheless, a moderate penalty score of 0.7 prevents disproportionate penalization for outputs that may still be valid but are slightly too short or long.

### 2.4. Weighting and Threshold Adjustments

Weights and override thresholds were adjusted iteratively based on transparency levels as follows:

High Transparency: $w_{c}$ = 0.50, $w_{s}$ = 0.30, $w_{l}$ = 0.20, threshold = 0.55

Moderate Transparency: $w_{c}$ = 0.60, $w_{s}$ = 0.30, $w_{l}$= 0.10, threshold = 0.65

Low Transparency: $w_{c}$ = 0.40, $w_{s}$ = 0.50, $w_{l}\;$= 0.10, threshold = 0.70

The override decision was computed as Equation (2):

(2) $$Override\;Decision=\left\{\begin{array}{cc} 1, & if\;S_{final}<Threshold\\ 0, & Otherwise.\;\;\;\;\;\;\;\;\;\;\;\;\;\;\;\;\;\;\; \end{array}\right.$$

Using the composite trust score, an override threshold above which the AI’s suggestion would be accepted and below which it would be overridden by the clinician was defined. These thresholds were empirically calibrated on the development set through iterative experimentation, initially setting conservative thresholds and then adjusting them upward or downward to achieve a balance where the number of necessary overrides (for incorrect AI suggestions) was minimized without unduly rejecting correct AI suggestions. Separate override thresholds were tuned for different transparency levels, with confidence as the dominant driver, followed by semantic similarity and length. For example, under *High Transparency* conditions (where the AI provides a clear rationale), a relatively lower confidence threshold was used to accept the AI’s recommendation (since the explanation adds trust), whereas under *Minimal Transparency*, a higher confidence threshold was required for acceptance. Because clinical decision-making hinges on understanding cues and their contextual salience, model confidence is treated as the most influential parameter; however, to guard against overconfidence, semantic similarity and length help objectivize the final decision in an interpretable way. During calibration, false acceptances were penalized more heavily than overrides to reflect clinical risk. Together, these choices yield an explicit, auditable rule whose behavior aligned with intended clinical use.

### 2.5. Statistical Analysis

Override rates were analyzed across multiple dimensions to assess the impact of AI Transparency Level, Confidence Calibration, and Diagnostic Similarity on clinician acceptance. An Overall Override Rate was determined as the proportion of AI-generated diagnoses that were overridden due to the final confidence-adjusted score falling below the predefined threshold. To evaluate the influence of explainability on clinician trust and decision-making, a separate analysis was conducted to examine the override rate by Transparency Level, categorized into three levels: low, moderate, and high with high confidence. High Confidence was added to the last level to preserve fidelity to the raw output and avoid subjective reclassification, as the model combined confidence and transparency in its self-assessment output. However, functionally, it represents cases where the AI claimed strong evidence and a clear reasoning path—essentially, high transparency with high diagnostic certainty.

To assess the effect of AI Confidence on acceptance rates, the Override Rate by Confidence was computed for three levels: 70–79%, 80–89%, and 90–99%. Finally, to explore the potential interaction effects between explainability and confidence, we calculated a combined Override Rate for both ranges.

A chi-square test was conducted to assess the association between transparency levels and override rates, with statistical significance set at *p* < 0.05. Additionally, pairwise post hoc comparisons between transparency groups were performed using the Tukey Honestly Significant Difference (HSD) test to identify specific differences between groups. Finally, Cramér’s V was calculated to assess the strength of association between categorical variables.

### 2.6. Ethics and Data Governance

This study was conducted in compliance with the Institutional Review Board (IRB) regulations governing the use of MIMIC-III data. All data were de-identified in accordance with the Health Insurance Portability and Accountability Act (HIPAA) guidelines. The research team completed the required CITI training on human subjects’ research before accessing the data. The study adhered to the principles of the Declaration of Helsinki.

## 3. Results

### 3.1. Overall Override Rate

The Overall Override rate across all AI-generated CVDs was 33.29% (2227 out of 6689 cases, non-override cases: 4462), indicating that approximately one-third of the diagnoses were overridden upon clinical review. This suggests moderate alignment between AI-generated outputs and clinician-verified diagnoses. This rate represents a substantial decrease from earlier iterations, where the initial override rate exceeded 87% before systematic parameter adjustments and model refinements were applied. The reduction in override rates reflects the impact of iterative tuning of AI confidence thresholds, similarity scoring, and transparency calibration. We present an example of the model’s running process in Supplementary Table S2.

### 3.2. Override Rates by Transparency Level

Our results showed an inverse relationship between Transparency Level and Override Rates, where the lower the transparency reported, the more likely the diagnosis was overridden. For diagnoses with Minimal Transparency, the override rate was 73.9% (99 out of 157 cases; 95% CI: 66.5–80.1%), reflecting limited explainability and increased clinician skepticism. Conversely, for diagnoses with Moderate Transparency, the override rate decreased to 49.3% (1631 out of 3307 cases; 95% CI: 47.6–51.0%), demonstrating that increased explainability improved clinician trust and reduced the frequency of overrides. The override rate further decreased to 14.9% (495 out of 3325 cases; 95% CI: 13.7–16.1%) for diagnoses with High Confidence, highlighting the critical role of transparency in enhancing AI trustworthiness. Given that the Transparency Level is generated by the LLM, the number of cases by level has an uneven distribution. This was a result of the model’s criteria based on the clinical findings, such as the presence or absence of supporting data or the ambiguity in the clinical records. This reflects a better representation of real-life clinical scenarios. Table 2 depicts the override rate across the transparency levels.

To assess whether clinician override rates vary by AI Transparency Level, we applied a Chi-Squared test using the contingency table shown in Table 3. With 2 degrees of freedom, the test yielded a χ^2^ of 991.01 and a *p*-value less than 0.0001, indicating a highly significant association between transparency level and override likelihood. Cramér’s V demonstrated a moderate effect size of 0.40. This means that clinicians overrode minimal transparency outputs more often and high-explainability outputs less often than expected under the null hypothesis of independence. This is presented in more detail in Table 3.

### 3.3. Override Rates by AI Confidence Ranges

**Figure 2:**
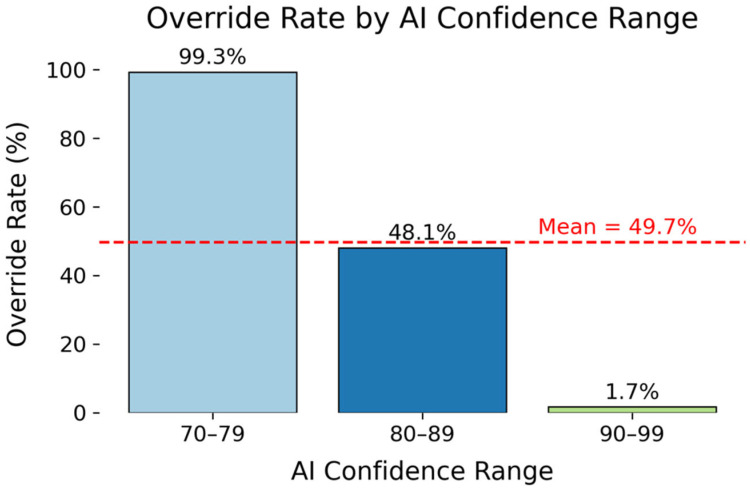
Bar plot showing Override Rate (%) by Confidence Range.

Override rates were examined across three AI confidence ranges (70–79%, 80–89%, 90–99%) to evaluate the impact of confidence calibration on override decisions. A similar indirect relationship between the level of confidence and the override rate was identified, with diagnoses more likely to be overridden with lower AI Confidence levels (Figure 2). For low-confidence predictions (70–79%), the override rate was 99.3% (147 out of 148 cases; 95% CI: 97.4–100%), reflecting near-universal rejection of low-confidence predictions. The rate decreased to 48.1% (2040 out of 4243 cases; 95% CI: 46.5–49.6%) when confidence ranged between 80% and 89%. The override rate ultimately decreased to 1.7% (390 out of 2298 cases; 95% CI: 1.4–2.4%) when the confidence level increased to 90–99%, showcasing near-complete clinical acceptance of high-confidence AI-generated diagnoses. Following a similar logic to Transparency Level, as confidence levels were assigned by the model, more cases fell in the 80–89% range, as the model tends to be conservative unless strong evidence exists. This results in a more realistic variation in diagnostic certainty. Table 4 shows the override rate across the confidence ranges.

### 3.4. Interaction Between Transparency and Confidence

Override rates were further stratified by both transparency level and confidence range to assess potential interaction effects. We identified a variable relationship between these two parameters, where confidence had a higher impact on override rates when it was lower. Specifically, when the model determined a low-confidence rate for its diagnoses, the override rate remained high across all Transparency levels. Conversely, when AI Confidence was moderate, the override rate decreased progressively, proportional to the Transparency Level, starting from 55.4% (31 out of 56 cases; 95% CI: 42.4–67.6%) for minimal explainability, to 49.9% (1591 out of 3182 cases; 95% CI: 48.1–51.6%) for moderate explainability, and 42.3% (448 out of 1059 cases; 95% CI: 39.4–45.3%) for high explainability with high confidence. AI Confidence, once more, was the most important determinant for override rates when it was high, with very low override rates. Meanwhile, the Transparency Level did not show a high effect as shown by an override rate of 0% with minimal explainability, 7.7% for moderate, and 1.4% for high explainability. Table 5 presents the interaction between the Transparency Level and Confidence Range and its influence on Override Rates.

When comparing groups across all nine combinations of transparency and confidence, the High Explainability + High Confidence with a 90–99% Confidence Range group showed the lowest override rate and consistently outperformed nearly every other combination. In pairwise tests, its mean override rate was significantly lower than that of all Minimal- and Moderate-transparency groups at both the 70–79% and 80–89% confidence levels (*p* < 0.001). Within the High Explainability condition itself, increasing confidence from 80–89% to 90–99% yielded a further significant drop in overrides, with a mean difference of approximately −0.41, and a *p*-value of less than 0.001.

The High Explainability + High Confidence with an 80–89% Confidence Range group likewise had significantly fewer overrides than the Minimal and Moderate groups at matching confidence ranges (*p* < 0.001), though it was outperformed by its own 90–99% counterpart. In contrast, comparisons among the Minimal and Moderate groups were mixed: some differences reached significance (e.g., Minimal Explainability with 70–79% Confidence vs. Minimal Explainability with 90–99% Confidence, Moderate Explainability with 70–79% Confidence vs. Moderate Explainability with 90–99% Confidence); however, no Minimal vs. Moderate pairing at the same confidence range was significant. These results suggest that providing full explanations *and* high model confidence, especially in the 90–99% range, produced the clearest reductions in clinician overrides, indicating that the combination of high explainability plus very high confidence is the most effective for minimizing overrides.

## 4. Discussion

By introducing a dynamic AI scoring framework to determine the quality of AI-decision support system responses, this study addresses one of the most significant impediments to the effective implementation of AI-decision support tools: low clinician trust due to uncertainty about their accuracy and reliability, while considering confidence calibrations to mitigate clinician overreliance [22,23]. The proposed framework significantly reduced clinician override rates from an initial 87.64% to 33.29%, highlighting substantial improvements in the alignment between AI-generated diagnoses and clinician assessments. These findings underscore the importance of adopting dynamic, multi-factorial approaches in AI-driven clinical decision support systems (CDSS) to enhance clinician trust and acceptance.

One of the most noteworthy outcomes of this research was the sharp reduction in overrides for high-confidence AI predictions (90–99%), with rates decreasing to just 1.7%. This result strongly supports the assertion that accurately calibrated confidence scores could significantly enhance clinician trust in AI recommendations. Comparable studies in the literature reinforce this perspective; for example, Guo et al. reported similar findings, demonstrating the critical importance of robust confidence calibration in reducing unnecessary clinician interventions and improving clinical workflow efficiency [24,25]. Our study extends these findings by explicitly integrating confidence calibration with transparency measures and semantic similarity scoring, demonstrating a comprehensive approach to enhancing clinician acceptance.

Conversely, diagnoses with lower AI confidence scores (70–79%) had an override rate of 99.3%, clearly indicating that clinicians’ near-universal rejection of uncertain predictions would be a common occurrence. This outcome highlights the need for careful calibration of AI systems, emphasizing the necessity of establishing context-specific confidence thresholds that clearly distinguish between trustworthy and unreliable predictions. Our findings corroborate recent literature underscoring the risk of false-positive diagnoses and unnecessary workload increases when AI confidence is inaccurately assessed or poorly communicated [26].

Transparency emerged as another critical factor influencing clinician acceptance of AI-driven diagnostic suggestions. Diagnoses characterized by moderate transparency levels had significantly lower override rates (49.3%) compared to those with minimal transparency (73.9%). These results align well with previous research by Ghassemi et al. and Rajkomar et al., which similarly found that increased AI transparency enhances clinician trust and promotes more effective integration of AI systems into clinical practice [27,28]. Our results further expand upon these studies by demonstrating the nuanced relationship between transparency and clinician overrides, particularly emphasizing the conditional importance of transparency depending on confidence levels.

Our analysis also uncovered a significant interaction between confidence and transparency. Specifically, transparency had a significant impact on clinician acceptance for predictions with moderate confidence scores (80–89%), whereas it had relatively little effect on predictions with high confidence scores (90–99%). This indicates that while transparency generally enhances clinician acceptance, the predominant factor driving clinician decision-making appears to be confidence calibration when confidence scores are very high. This nuanced finding emphasizes the importance of simultaneously addressing both transparency and confidence calibration, tailored to the specific contexts in which AI-DSS are deployed.

The reduction in override rates not only reflects improved model accuracy but also has direct implications for clinical workflow efficiency and patient safety. High override rates often translate into increased clinician workload and cognitive fatigue, introducing variability and risk of human error [24]. By contrast, reducing override rates through improved model calibration and transparency allows clinicians to trust AI recommendations more frequently, decreasing the need for constant intervention [29]. This, in turn, reduces diagnostic delays, minimizes error-prone revisions, and enhances the consistency and reliability of clinical decisions, ultimately leading to better patient outcomes [30].

Dynamically adjusting the acceptance threshold also carries governance and medico-legal implications because it changes the clinical significance of the system’s output and the degree of reasonable reliance by clinicians. To remain within accepted boundaries for clinical decision support, the basis for recommendations and the gating logic should be reviewable by users, with versioned procedures and auditable logs capturing the model’s confidence, explanation, similarity score, the applied threshold, and the final decision [31,32]. Risk frameworks for software as a medical device emphasize that changes that tighten or loosen acceptance criteria alter the risk profile and therefore warrant controlled change management, pre-release verification, and post-market monitoring [33]. From a liability perspective, documenting why a recommendation was accepted or overridden supports the physician’s learned-intermediary role; adopting a conservative posture, such as raising the bar when transparency is low, reduces the chance of over-reliance while preserving clinical judgment [34,35]. In jurisdictions implementing the EU AI Act, many health AI systems will be subject to requirements for risk management, logging, user transparency, and human oversight, obligations that align with explicit thresholds, visible rationales, immutable logs, and routine drift audits described here [36].

## 5. Strengths and Limitations

Our framework offers several practical implementations. Its modular and adaptable design supports broad generalizability across diverse clinical domains beyond cardiovascular diagnoses, such as oncology, neurology, and infectious diseases [37,38,39]. By embedding transparency and confidence calibration directly into clinical workflows, this framework can potentially streamline decision-making processes, reduce clinician workload, and enhance patient outcomes across various healthcare settings. Moreover, integration into existing electronic health records (EHR) systems could further facilitate the practical implementation, providing clinicians with real-time, actionable insights that align closely with clinical practice realities [28].

As the framework is work-flow agnostic, it can be tuned for these other domains with targeted modifications, such as replacing plain text similarity with ontology-aware similarity (e.g., SNOMED CT/ICD-10/LOINC mapping with synonym/hypernym expansion) so that semantic similarity reflects domain-salient concordance (e.g., site–histology–stage in oncology; organism–source–severity in infectious disease; focal deficit–imaging–onset tempo in neurology). The transparency rubric can be redefined to require evidence appropriate to the target domain while capping its influence so it cannot compensate for low confidence or similarity. Additionally, the parameter weights and transparency-specific thresholds can be recalibrated based on the different specialties’ terminologies to preserve penalization of false acceptances. Confidence can change with a different domain, requiring tailored confidence calibration. Finally, out-of-distribution guardrails that force manual review when evidence is sparse or contradictory can be added and validated prospectively, logging explanations and ratings for drift audits and subgroup analyses. This preserves the conservative risk posture (higher acceptance thresholds when transparency is low) while adapting decision criteria to domain context.

However, several methodological and practical limitations must be acknowledged. First, the transparency measure utilized relied on self-rated assessments by the AI model rather than direct human expert evaluations. Although providing a standardized proxy for explainability in the current experiment, this approach may introduce biases or inconsistencies in transparency ratings, potentially limiting the interpretability of our findings related to transparency. Considering this, we recognize that it is paramount that in future research we should incorporate expert-driven transparency assessments to validate and potentially refine these findings. This will be achieved in future studies where we will compare the model’s override rates with real-time physician override rates.

Additionally, the retrospective design using the MIMIC-III dataset poses inherent limitations regarding the generalizability and real-world applicability of results. The dataset’s retrospective nature restricts direct insights into prospective clinical settings where real-time decision-making dynamics and clinical workflows may significantly differ. Consequently, we will confirm the framework’s utility in future prospective studies across diverse clinical environments and patient populations to refine its parameters further.

The complexity of the proposed scoring system also needs to be considered. While the sophisticated integration of confidence calibration, transparency, and semantic similarity scoring has proven effective in enhancing clinical acceptance, its practical implementation in real-time clinical workflows may pose challenges. High model complexity could potentially increase computational requirements and decision-making latency, highlighting the trade-off between model sophistication and practical usability. While this study focused on algorithmic performance rather than response timing, future studies will explicitly address these concerns by assessing the feasibility of real-time integration and exploring simplified implementations that retain predictive reliability without excessive computational burdens.

Furthermore, the domain-specific bias introduced by training and validating the model exclusively on cardiovascular data from the MIMIC-III dataset could limit generalizability. The population demographics, clinical practices, and diagnostic conventions specific to the dataset may not accurately reflect broader clinical environments, potentially impacting the framework’s performance when applied outside its original validation context. Although the model can be tailored for different specialties and diseases, as described previously, future research should prioritize multi-institutional validation studies leveraging diverse patient populations and clinical settings to assess the generalizability and robustness of this framework rigorously.

Finally, as this first model’s implementation was intended as a proof-of-concept to demonstrate that the dynamic framework worked and to reflect how the different levels of confidence and transparency affected the override rates, future directions will evaluate real-time adaptive calibration based on ongoing clinician feedback. Continuous iterative refinement, informed by direct clinical inputs, could significantly enhance the predictive accuracy and clinical relevance of AI-driven diagnostic recommendations. Real-time adaptation could effectively address edge cases and rare diagnostic scenarios that current static calibration approaches might inadequately capture.

## 6. Conclusions

This study applied the proposed framework to 6689 cardiovascular cases from MIMIC-III. By combining model-reported confidence, semantic similarity between AI-generated and clinician-verified diagnoses, a length-based score, and transparency-conditioned thresholds, the current framework substantially reduced overrides relative to earlier iterations. The analysis showed a strong, graded association between confidence and model acceptance, with markedly lower overrides at higher confidence levels, and a consistent reduction when transparency improved (73.9% with minimal transparency vs. 49.3% with moderate). When both transparency and confidence were high, override rates were the lowest across strata. These results, supported by significance testing and a moderate effect size, indicate that calibrating acceptance on confidence while allowing transparency to tighten the threshold, and using similarity and length to discourage vague or list-like outputs, yields an explicit, auditable rule that has the potential to better align AI suggestions with clinician judgment and reduce unnecessary interventions.

The next steps should focus on prospective clinical validation and real-time integration. Priorities include confirming performance in live workflows, assessing latency and usability, and incorporating expert review to validate and, if needed, refine the transparency signal. Because terminology and documentation patterns vary across specialties, the same acceptance logic can be tuned for other domains by adapting similarity to domain ontologies, re-estimating weights and thresholds on small development sets, and applying confidence calibration where needed. Integration within electronic health records, simple guardrails for out-of-distribution or contradictory evidence, and routine drift audits will help maintain reliability and generalizability across sites and populations, while preserving the conservative posture of raising the acceptance bar when explanations are weaker.

## Figures and Tables

**Table 1:** Cardiovascular conditions included in *diagnoses_icd* with corresponding ICD-9 code ranges.

Conditions	ICD-9 Code
Ischemic heart disease	410–414
Hypertension	401–405
Cerebrovascular Disease	430–438
Valvular heart diseases	394–397
Other Heart Diseases	420–429

**Table 2:** Override Rate by Transparency Level.

Transparency Level	Override Rate (%)	Count
Minimal	73.9	157
Moderate	49.3	3307
High Confidence	14.9	3225

**Table 3:** Contingency tables for the Chi-Square Test of Independence, comparing the results obtained with the proposed framework (above) to the expected counts under independence (below) (χ^2^ = 991.01, *p* < 0.001, Cramér’s V = 0.4).

**Transparency Level**	**Not Overridden (*n*)**	**Overridden (*n*)**
Minimal	41	116
Moderate	1676	1631
High Confidence	2745	480
**Transparency Level**	**Not Overridden (Expected)**	**Overridden (Expected)**
Minimal	104.73	52.27
Moderate	2205.99	1101.15
High Confidence	2151.29	1071.71

**Table 4:** Override Rates by AI Confidence Ranges.

Confidence Range	Override Rate (%)	Count
70–79	99.3	148
80–89	48.1	4243
90–99	1.7	2298

**Table 5:** Interaction between Transparency Levels and Confidence Ranges. These findings suggest that confidence and transparency exert complementary effects on clinical acceptance of AI-generated diagnoses. While low-confidence predictions were universally rejected, the combination of high confidence and transparency was associated with significantly improved acceptance rates.

Transparency Level	Confidence Range	Override Rate (%)	Count	95% CI (%)
Minimal	70–79	100.0	85	95.6–100
	80–89	55.4	56	42.4–67.6
	90–99	0.0	16	0–19.4
Moderate	70–79	98.4	62	94.1–100
	80–89	49.9	3182	48.1–51.6
	90–99	7.7	51	5.1–15.4
High Explainability + High Confidence	70–79	100.0	1	20.6–100
	80–89	42.3	1059	39.4–45.3
	90–99	1.4	2165	1.0–2.0

## Data Availability

The raw data supporting the conclusions of this article will be made available by the authors on request.

## Supplementary Materials

The following supporting information can be downloaded at: https://www.mdpi.com/article/10.3390/diagnostics15172204/s1, Table S1: Complete list of cardiovascular diseases and ICD-9 codes; Table S2: Example demonstration of the model’s working process.

## References

[1] Gomez-Cabello C.A., Borna S., Pressman S., Haider S.A., Haider C.R., Forte A.J. (2024). Artificial-Intelligence-Based Clinical Decision Support Systems in Primary Care: A Scoping Review of Current Clinical Implementations. Eur. J. Investig. Health Psychol. Educ..

[2] Ouanes K., Farhah N. (2024). Effectiveness of Artificial Intelligence (AI) in clinical decision support systems and care delivery. J. Med. Syst..

[3] Kelly C.J., Karthikesalingam A., Suleyman M., Corrado G., King D. (2019). Key challenges for delivering clinical impact with artificial intelligence. BMC Med..

[4] Rosenbacke R., Melhus A., McKee M., Stuckler D. (2024). How Explainable Artificial Intelligence Can Increase or Decrease Clinicians’ Trust in AI Applications in Health Care: Systematic Review. JMIR AI.

[5] Pias T.S., Afrose S., Tuli M.D., Trisha I.H., Deng X., Nemeroff C.B., Yao D.D. (2025). Low responsiveness of machine learning models to critical or deteriorating health conditions. Commun. Med..

[6] Lindenmeyer A., Blattmann M., Franke S., Neumuth T., Schneider D. (2024). Inadequacy of common stochastic neural networks for reliable clinical decision support. arXiv.

[7] Lindenmeyer A., Blattmann M., Franke S., Neumuth T., Schneider D. (2025). Towards Trustworthy AI in Healthcare: Epistemic Uncertainty Estimation for Clinical Decision Support. J. Pers. Med..

[8] Rezaeian O., Bayrak A.E., Asan O. (2025). Explainability and AI Confidence in Clinical Decision Support Systems: Effects on Trust, Diagnostic Performance, and Cognitive Load in Breast Cancer Care. arXiv.

[9] Tahir D. Health Care AI, Intended to Save Money, Turns Out to Require a Lot of Expensive Humans. https://www.hcinnovationgroup.com/analytics-ai/news/55263070/experts-healthcare-ai-will-require-expensive-humans.

[10] Scott I.A., van der Vegt A., Lane P., McPhail S., Magrabi F. (2024). Achieving large-scale clinician adoption of AI-enabled decision support. BMJ Health Care Inform..

[11] Kessler D., Zhu M., Gregory C.R., Mehanian C., Avila J., Avitable N., Coneybeare D., Das D., Dessie A., Kennedy T.M. (2024). Development and testing of a deep learning algorithm to detect lung consolidation among children with pneumonia using hand-held ultrasound. PLoS ONE.

[12] Kuroda Y., Kaneko T., Yoshikawa H., Uchiyama S., Nagata Y., Matsushita Y., Hiki M., Minamino T., Takahashi K., Daida H. (2023). Artificial intelligence-based point-of-care lung ultrasound for screening COVID-19 pneumoniae: Comparison with CT scans. PLoS ONE.

[13] Gulshan V., Peng L., Coram M., Stumpe M.C., Wu D., Narayanaswamy A., Venugopalan S., Widner K., Madams T., Cuadros J. (2016). Development and Validation of a Deep Learning Algorithm for Detection of Diabetic Retinopathy in Retinal Fundus Photographs. JAMA.

[14] Esteva A., Kuprel B., Novoa R.A., Ko J., Swetter S.M., Blau H.M., Thrun S. (2017). Dermatologist-level classification of skin cancer with deep neural networks. Nature.

[15] Li F., Xin H., Zhang J., Fu M., Zhou J., Lian Z. (2021). Prediction model of in-hospital mortality in intensive care unit patients with heart failure: Machine learning-based, retrospective analysis of the MIMIC-III database. BMJ Open.

[16] Su D., Zheng J., Shao Y.-K., Liu J.-Y., Liu X.-X., Yu K., Feng B.-H., Mei H., Qin S. (2025). Developing and validating a machine learning-based model for predicting in-hospital mortality among ICU-admitted heart failure patients: A study utilizing the MIMIC-III database. Digit. Health.

[17] Karboub K., Tabaa M. (2022). A machine learning based discharge prediction of cardiovascular diseases patients in intensive care units. Healthcare.

[18] Kong G., Lin K., Hu Y. (2020). Using machine learning methods to predict in-hospital mortality of sepsis patients in the ICU. BMC Med. Inform. Decis. Mak..

[19] Cui W., Finkelstein J. (2025). Leveraging LLMs for Early Diagnosis in the Emergency Department: Comparing ClinicalBERT and GPT-4. Stud. Health Technol. Inform..

[20] Johnson A.E., Pollard T.J., Shen L., Lehman L.W., Feng M., Ghassemi M., Moody B., Szolovits P., Celi L.A., Mark R.G. (2016). MIMIC-III, a freely accessible critical care database. Sci. Data.

[21] Cer D., Yang Y., Kong S.-Y., Hua N., Limtiaco N., John R.S., Constant N., Guajardo-Cespedes M., Yuan S., Tar C. (2018). Universal sentence encoder. arXiv.

[22] Amann J., Blasimme A., Vayena E., Frey D., Madai V.I., Precise Q.C. (2020). Explainability for artificial intelligence in healthcare: A multidisciplinary perspective. BMC Med. Inform. Decis. Mak..

[23] Amann J., Vetter D., Blomberg S.N., Christensen H.C., Coffee M., Gerke S., Gilbert T.K., Hagendorff T., Holm S., Livne M. (2022). To explain or not to explain?-Artificial intelligence explainability in clinical decision support systems. PLoS Digit. Health.

[24] Olakotan O.O., Mohd Yusof M. (2021). The appropriateness of clinical decision support systems alerts in supporting clinical workflows: A systematic review. Health Inform. J..

[25] Guo C., Pleiss G., Sun Y., Weinberger K.Q. On calibration of modern neural networks. Proceedings of the International Conference on Machine Learning.

[26] Topol E.J. (2019). High-performance medicine: The convergence of human and artificial intelligence. Nat. Med..

[27] Ghassemi M., Oakden-Rayner L., Beam A.L. (2021). The false hope of current approaches to explainable artificial intelligence in health care. Lancet Digit. Health.

[28] Rajkomar A., Dean J., Kohane I. (2019). Machine Learning in Medicine. N. Engl. J. Med..

[29] Lee D., Yoon S.N. (2021). Application of Artificial Intelligence-Based Technologies in the Healthcare Industry: Opportunities and Challenges. Int. J. Environ. Res. Public Health.

[30] Bright T.J., Wong A., Dhurjati R., Bristow E., Bastian L., Coeytaux R.R., Samsa G., Hasselblad V., Williams J.W., Musty M.D. (2012). Effect of clinical decision-support systems: A systematic review. Ann. Intern. Med..

[31] U. S. Food and Drug Administration (2022). Clinical Decision Support Software—Guidance for Industry and FDA Staff.

[32] U. S. Food and Drug Administration (2024). Clinical Decision Support Software: Frequently Asked Questions (FAQs).

[33] International Medical Device Regulators Forum (2014). “Software as a Medical Device”: Possible Framework for Risk Categorization and Corresponding Considerations (IMDRF/SaMD WG/N12FINAL:2014). https://www.imdrf.org/sites/default/files/docs/imdrf/final/technical/imdrf-tech-140918-samd-framework-risk-categorization-141013.pdf.

[34] Mello M.M., Guha N. (2024). Understanding Liability Risk from Using Health Care Artificial Intelligence Tools. N. Engl. J. Med..

[35] Price W.N., Gerke S., Cohen I.G. (2019). Potential Liability for Physicians Using Artificial Intelligence. JAMA.

[36] European Union (2024). Regulation (EU) 2024/1689 of the European Parliament and of the Council of 13 June 2024 Laying Down Harmonised Rules on Artificial Intelligence (Artificial Intelligence Act).

[37] Kalani M., Anjankar A. (2024). Revolutionizing Neurology: The Role of Artificial Intelligence in Advancing Diagnosis and Treatment. Cureus.

[38] McKinney S.M., Sieniek M., Godbole V., Godwin J., Antropova N., Ashrafian H., Back T., Chesus M., Corrado G.S., Darzi A. (2020). International evaluation of an AI system for breast cancer screening. Nature.

[39] Sarantopoulos A., Mastori Kourmpani C., Yokarasa A.L., Makamanzi C., Antoniou P., Spernovasilis N., Tsioutis C. (2024). Artificial Intelligence in Infectious Disease Clinical Practice: An Overview of Gaps, Opportunities, and Limitations. Trop. Med. Infect. Dis..

