# Supplementary material for: Enhancing Clinician Trust in AI Diagnostics: A Dynamic Framework for Confidence Calibration and Transparency
Source: Diagnostics (Basel). 2025 Aug 30;15(17):2204. doi: 10.3390/diagnostics15172204 (PMC12428550; doi:10.3390/diagnostics15172204)
Supplement: Supplementary file 1 [file diagnostics-15-02204-s001.zip › diagnostics-3765142-supplementary.pdf]

ICD-9 Code	Diagnosis Description
401	Essential hypertension
402	Hypertensive heart disease
403	Hypertensive renal disease
404	Hypertensive renal and heart disease
405	Secondary hypertension
410	Acute myocardial infarction
411	Other acute and subacute forms of ischemic heart disease
412	Old myocardial infarction
413	Angina pectoris
414	Other forms of chronic ischemic heart disease
420	Acute pericarditis
421	Acute and subacute endocarditis
422	Acute myocarditis
423	Other diseases of pericardium
424	Other diseases of endocardium
425	Cardiomyopathy
426	Conduction disorders
427	Cardiac dysrhythmias
428	Heart failure
429	Ill-defined descriptions and complications of heart disease
430	Subarachnoid hemorrhage
431	Intracerebral hemorrhage
432	Other and unspecified intracranial hemorrhage
433	Occlusion and stenosis of precerebral arteries
434	Occlusion of cerebral arteries
435	Transient cerebral ischemia
436	Acute, but ill-defined, cerebrovascular disease
437	Other and ill-defined cerebrovascular disease
438	Late effects of cerebrovascular disease

Supplementary Table S1. Complete list of cardiovascular diseases and ICD-9 codes.

Part I: metadata of the running process:
**LLM Diagnosis System running log: ai_diagnosis_20250321_184745.log**<br>**LLM Model**: qwen2.5:latest<br>MIMIC-III Database connection successful.<br>Saved 6689 cases to cache: mimic_data_cardiovascular_cache.parquet<br>Loaded 6689 rows from MIMIC-III.<br>DataProcessor initialized in PRODUCTION mode<br>Loading data from cache: mimic_data_cardiovascular_cache.parquet<br>Limited to 6689 cases from cache<br>Successfully loaded 6689 cases from cache<br>Processing full dataset with 6689 rows<br>Columns in the DataFrame: ['Subject_ID', 'Symptoms', 'Real_Diagnosis']
**Part II: Diagnosis from MIMIC-III Data**
**Real_Diagnosis**: Anterior Myocardial Infarction<br>**Confidence**: 95<br>**Transparency Level**: High Confidence
**Part III: AI (LLM) Response:**
**Diagnosis**: Anterior Myocardial Infarction<br>**Confidence**: 95<br>**Transparency Level**: High Confidence<br>**Explanation (by LLM):** The diagnosis of anterior myocardial infarction is strongly supported by the presence of symptoms (chest pain), ECG findings (anterior MI), and elevated troponin levels. The patient also underwent treatment for myocardial infarction, including hospitalization, medication management, and follow-up appointments with a cardiologist. These factors provide high confidence in the diagnosis.<br>**Raw Transparency Level**: 'High Confidence'<br>**Normalized Transparency Level**: 'High Confidence'<br>Checking if 'High Confidence' is in valid transparency levels: {'Low', 'Moderate', 'High Confidence', 'High', 'Very High', 'Minimal', 'Very Low'}<br>**Parsed response → Diagnosis**: Anterior Myocardial Infarction, Confidence: 95, Transparency: High Confidence<br>**Parsed → Dx**: Anterior Myocardial Infarction | Conf: 95 | Trans: High Confidence<br>**AI Diagnosis**: anterior myocardial infarction<br>**Jaccard**: 0.00, Levenshtein: 0.39, Final: 0.39
**Part IV: Decision**
**Decision**: Accept<br>**Final Score**: 0.78 vs Threshold: 0.65<br>**AI Diagnosis**: Anterior Myocardial Infarction<br>**Scores**: {'confidence': 0.8333333333333334, 'similarity': 0.39, 'length': 1.0}<br>**Transparency Level**: Moderate

Supplementary Table S2. Example demonstration of the model’s working process.
